# Long-Term Reproducibility of BMD-Measurements with Clinical QCT Using Simultaneous and Asynchronous Calibration Methods and Different Measurement and Reconstruction Protocols

**DOI:** 10.1007/s00223-024-01303-3

**Published:** 2024-10-16

**Authors:** Sophie du Mont, Reinhard Barkmann, Timo Damm, Jaime Peña, Stefan Reinhold, Marcus Both, Meike Mainusch, Claus-Christian Glüer

**Affiliations:** 1 Section Biomedical Imaging, Department of Radiology and Neuroradiology, University Medical Center Schleswig-Holstein (UKSH), Kiel University, Kiel, Germany; 2 Department of Computer Science, Multimedia Information Processing Group, Kiel University, Kiel, Germany; 3 Department of Radiology and Neuroradiology, University Medical Center Schleswig-Holstein (UKSH), Kiel University, Kiel, Germany

**Keywords:** Bone densitometry, Quantitative computed tomography, Asynchronous calibration methods, Osteoporosis, European spine phantom

## Abstract

Osteoporosis is underdiagnosed and undertreated. To improve timely fracture risk assessment optimized densitometry methods are required such as opportunistic spinal quantitative computed tomography (QCT). However, it is unclear how to best calibrate these scans and correct for potential scanner drift of QCT when used for monitoring bone mineral density (BMD) changes. We compared gold standard simultaneous calibration with asynchronous calibration methods, assessing mid-term (12 weeks) and long-term (1.5 years) reproducibility of BMD measurements. Cortical and trabecular compartments of the European Spine Phantom were studied with ten different protocols including low dose and high resolution (HR)-modes. Based on weekly phantom data, we compared simultaneous calibration to asynchronous single (termed global) or monthly calibration. The accuracy was better for trabecular measurements than for cortical measurements for all calibration methods. Reproducibility was excellent for all methods and slightly better for asynchronous than for simultaneous calibration both for trabecular and cortical bone. For HR protocols, reproducibility was better than for low dose measurements. In trabecular compartments averaged HR-BMD remained stable for global (− 0.1%/year, ns) but not for simultaneous calibration (− 1.5%/year, *p* < 0.001). No significant drifts could be detected for averaged low dose BMD (− 0.9 to + 0.8%/year) for either calibration method. Our data suggest that with regard to precision and accuracy measurements with asynchronous calibration are suitable for vertebral BMD assessment (no contrast agents) in clinical practice. Regular (e.g., monthly) stability tests using a calibration phantom could assure long term stability of at least 1 year.

**Supplementary Information:**

The online version contains supplementary material available at 10.1007/s00223-024-01303-3.

## Introduction

Osteoporosis is a systemic musculoskeletal disease which is defined by the reduction of bone mass and alteration of the bone’s microstructure leading to an increased fracture risk [1]. Odén et al. estimate that between the year 2010 and 2040 the number of individuals, which carry a high risk for osteoporotic fractures, will be doubled worldwide [2]. Osteoporosis-related fractures (e.g., vertebral fractures, and fractures of the hip or wrist) do not only cause a major impairment for the patient but cause medical costs of over $17 billion annually alone in the US [3]. Therefore, timely identification of patients at enhanced risk of fractures is essential to initiate appropriate therapeutic options in the disease’s early stages. However, osteoporosis remains underdiagnosed and undertreated today [4, 5]. Bone densitometry assessment with quantitative computed tomography (QCT) allows 3-dimensional measurements of volumetric BMD (in mg/cm^3^) and represents an alternative to the established dual X-ray absorptiometry (DXA with results in areal BMD in g/cm^2^) method [6]. Conventionally, for BMD-measurement with clinical QCT a simultaneous calibration with a calibration phantom placed underneath the patient’s spine is required in order to transform CT’s attenuation (in Hounsfield-Units) into BMD using a linear regression model [7]. Millions of QCT scans are performed annually for indications other than osteoporosis. By means of automated analysis patients at enhanced risk of fracture could be detected, so called *opportunistic screening*. However, HU may differ substantially across scanner types and manufacturers and without calibration standardized risk assessment thus can be affected by accuracy errors. Recent advancements in CT technology enabled the introduction of a BMD measurement with asynchronous calibration methods, i.e., measurements of a calibration standard at regular time intervals (i.e., monthly), obviating the need to measure a calibration phantom underneath each patient scanned, as needed for simultaneous calibration. Opportunistic screening [6] would reduce radiation exposure for the patient as fewer imaging procedures are needed, also lowering healthcare costs [8]. Recent studies indicate good clinical results for asynchronous calibrations for BMD screening applications. Those studies show that BMD values from routine abdominal or pelvic CT scans can be used for fracture prediction [9–13]. Moreover, recent findings suggest that simultaneous calibration may add a phantom-induced bias [11] and perhaps cause additional imaging noise. Furthermore, it is unclear how often calibration measurements for asynchronous calibration need to be repeated to assure timely detection of drifts of malfunctioning CT scanners and whether long term stability of modern CT equipment would be sufficient to use asynchronous calibration for monitoring BMD changes over time. For these aspects, precision and accuracy of asynchronous calibration should be assessed with a long-term view and for both the cortical and trabecular compartments—recent studies mainly focused on measurements of the trabecular compartments [11, 13].

The objective of this study was to compare the performance of asynchronous calibration to the current gold standard of dedicated QCT measurements with simultaneous calibration by investigating mid- and long-term precision and whether equipment drifts could affect BMD measurements of the two calibration methods. Long-term stability cannot be assessed accurately in patients since their BMD may drift over time and radiation doses would be prohibitive. Therefore, we took measurements on the European Spine Phantom, an established standard for quality assurance in osteoporosis. This also permitted to study precision and drifts for a variety of different reconstruction protocols extended over a long-term period of 1.5 years, typical of follow up time intervals of patients.

## Materials and Methods

All measurements have been conducted in the context of the multicentric ‘*DIAGNOSTIK-BILANZ’*-study, including 10 centres for recruitment and CT-measurements, organized by the Molecular Imaging North Competence Centre, a department of Radiology and Neuroradiology, University Medical Centre Schleswig–Holstein (UKSH), Kiel University, Kiel, Germany. Volunteers included were patients on long-term bisphosphonate treatment (at least the last 4 years). Spinal CT-measurements were performed with two different protocols of QCT, a high-resolution protocol for enhanced depiction of the trabecular vertebral structure (HR-QCT) and a low dose QCT-protocol to enable density measurements on larger part of the spine compared to standard QCT, which usually is applied to the lumbar spine only.

All patient measurements (data not included in this publication) were performed on the vertebrae simultaneously with a calibration phantom (*Bone Density Calibration Phantom, BDC-Phantom)* being placed below the spine. In Kiel, a similar setup using the BDC-Phantom together with a phantom simulating human vertebra (European Spine Phantom, ESP)* (*both phantoms: QRM GmbH, Moehrendorf, Germany) were used for weekly measurements over a total period of 14 months (27.07.2016–12.09.2017). After the first 12 weeks of measuring, a 9 months’ lasting recess was implemented in the testing process followed by a second measurement period of 12 weeks. We implemented a recess between two measurement periods in order to imitate a typical clinical set-up in which the patient re-visits his/her physician for additional scanning. During every individual measurement the ESP was placed on the BDC-Phantom to simulate a patient’s scan (Fig. S1, Appendix). This paper is based on measurements obtained on the ESP.

The ESP consists of three artificial vertebrae with nominal trabecular hydroxyapatite densities of 50 (in L1), 100 (in L2) and 200 (in L3) mg HA/cm^3^ and nominal cortical densities of 800 mg/cm^3^ for each vertebra surrounded by tissue mimicking material (Fig. S1). Cortical thickness increases from L1 to L3 [14]. Measured BMD values for ESP for trabecular compartments as stated in an acceptance-test-protocol by QRM GmbH are 50.5 mg HA/cm^3^ (L1), 101.2 mg HA/cm^3^ (L2), L3 200.6 mg HA/cm^3^ (L3) [15]. The BDC-Phantom comprises three parallel rods with hydroxyapatite densities of 100.0, 0.0 and 200.0 mg/cm^3^ [16]. Exact values for the phantom used in Kiel were 99.5 mg/cm^3^, 0 mg/cm^3^ and 203.0 mg HA /cm^3^.

All measurements were performed with the *SOMATOM Sensation 64* (Siemens AG, Erlangen, Germany), a 64-multislice CT scanner, using two different scanning protocols: a high-resolution protocol and a low dose protocol (Table S1, Appendix). The HR was run with the B70s kernel, increment 0.3 mm and slickness 0.6 mm. Low dose images can be acquired and reconstructed with different reconstructions kernels, increments and slice thickness. Since these protocols can also affect accuracy and precision, we studied three different bone-kernels (B40s, B60s, and B80s) with a range of different combinations of increments and slice thickness values (Table S1, Appendix). While ‘hard’ kernels (e.g., B80s) produce a sharp image with comparatively high noise value, ‘soft’ kernels (B40s) create images with a lower spatial resolution but less noise [7, 17]. The HR protocol was set up to enable an additional measurement of trabecular and cortical structure parameters in patients. Although structural assessment of bone is not relevant in this study due to the lack of interior structures in the ESP, sharper images with higher resolution can be achieved combined with less noise due to the higher radiation dose. For the low dose protocol, nine different reconstructions were used to cover a wider range of possible applications of the method (Table 1). The low dose protocol was implemented in the patient study to enable a scanning of a larger part of the spine with still acceptable radiation dose. For the high-resolution protocol, a tube current of 120 kV, a tube current time product of 355 mAs and a field of view (FoV) of 80 mm was chosen. For the low dose protocol, a tube current of 80 kV, a tube current time product of 120 mAs and a FoV of 200 mm was used.

**Table 1:** Midterm precision and mean long-term precision (for ESP vertebrae L1–L3, in %) for trabecular and cortical bone with simultaneous and asynchronous (global) calibration methods for all reconstruction kernels and monthly calibration for B40s, 1.0 mm slice thickness

	Trabecular bone				Cortical bone			
	Sim. Cal		Asynchr. Cal		Sim. Cal		Asynchr. Cal	
	Mid-term prec	Long-term prec	Mid-term prec	Long-term prec	Mid-term prec	Long-term prec	Mid-term prec	Long-term prec
B70s, 0.6 mm	0.80	1.01	0.34	0.43	0.61	3.36	0.20	1.08
B40s, 0.6 mm	2.59	3.12	2.00	2.42	0.99	4.44	0.35	1.55
B40s, 1.0 mm	2.86	3.44	1.60	1.92	0.67	3.01	0.34	1.51
B40s, 3.0 mm	2.31	2.82	1.70	2.05	1.44	6.45	1.39	6.28
B60s, 0.6 mm	3.76	5.14	1.75	2.36	1.96	10.48	0.38	1.98
B60s, 1.0 mm	3.01	3.80	1.63	2.02	1.89	10.06	0.39	2.04
B60s, 3.0 mm	3.48	4.43	1.65	2.04	2.48	13.23	2.08	10.99
B80s, 0.6 mm	2.82	3.96	1.35	1.99	4.01	27.40	0.36	2.45
B80s, 1.0 mm	3.41	4.22	1.42	1.78	3.08	20.92	0.41	2.84
B80s, 3.0 mm	3.42	4.18	2.03	2.50	3.03	20.85	1.60	11.15
B40s, 1.0 mm (monthly)	–	–	2.02	2.45	–	–	1.40	6.20

Image processing was conducted with the software package Structural*Insight* to calculate BMD from CT attenuation values. This imaging processing software was developed by our section and combines all important quantitative CT data processing steps including quality assurance, calibration, threshold segmentation and analysis [18]. Intermediate application of a mask, created especially for these artificial vertebrae, helped to find a well-defined region prior to thresholding (Fig. S2, Appendix).

The ESP was scanned on top of the BDC, permitting analysis with simultaneous calibration. In the first analysis the densities of the artificial vertebrae of the ESP were calibrated using the densities of the compartments of the BDC, which was scanned at the same time (simultaneous calibration). To simulate an asynchronous (global) calibration uncalibrated data were calibrated by using one mean calibration curve only, calculated from the average CT-values of the BDC from all measurements (one curve for HR protocol and one for all low dose protocols). Analogous to the asynchronous (global) calibration for the asynchronous (monthly) calibration (only applied onto datasets with kernel B40s, 1.0 mm slice thickness) the first synchronous calibration curve of each month was used for calibrating all following datasets of this particular month. Secondly all datasets were processed using threshold segmentation. As depicted in figure S2 (appendix) narrower segmentation masks were placed in transverse planes in the middle of the vertebrae due to the problem that in low resolution scans with thick layers (e.g., 3.0 mm) inferior and superior endplates of adjacent vertebral bodies could not be differentiated thus leading to segmentation errors. By using a narrower segmentation mask threshold segmentation for thick layers remained possible. As a result, only vertical parts of the cortex of each ESP vertebra were analysed.

### Statistical Analysis

**Fig. 1:**
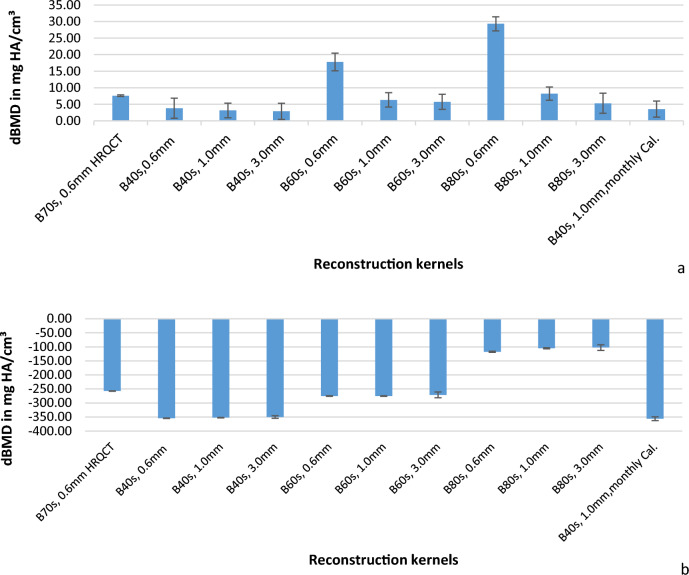
**a, b** Bars depict accuracy errors (deviation of mean BMD from standard ESP values) and error bars depict mean mid-term precision errors (for vertebrae L1–L3) for trabecular bone (*) (**a**) and cortical bone (**b**) with asynchronous (global) calibration for all reconstruction kernels and monthly calibration for B40s, 1.0 mm slice thickness, (*for trabecular compartments nominal BMD values according to the QRM acceptance-test were used)

**Fig. 2:**
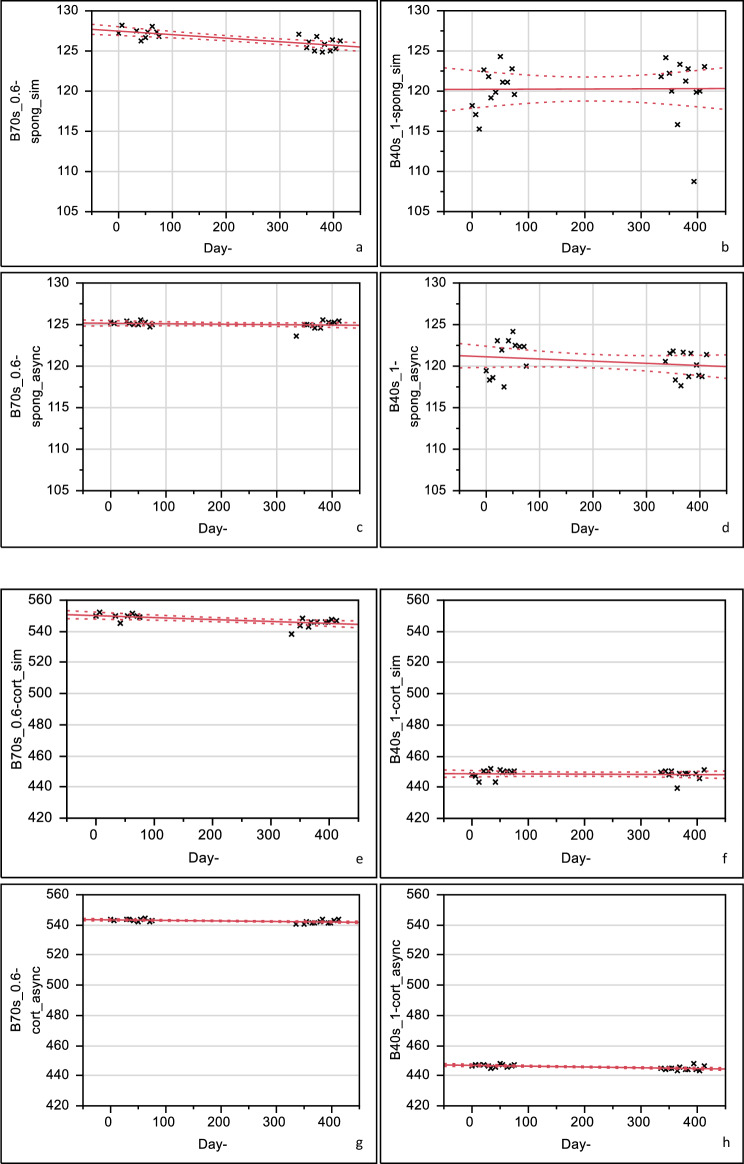
**a–d** *Trabecular* BMD-values over time for simultaneous (top) and global (bottom) calibrations with reconstruction kernels B70s, 0.6 mm and B40s, 1.0 mm slice thickness and monthly calibration with B40s, 1.0 mm slice thickness. **e–h** *Cortical* BMD-values over time for simultaneous and global calibrations with reconstruction kernels B70s, 0.6 mm and B40s, 1.0 mm slice thickness and monthly calibration with B40s, 1.0 mm slice thickness

The statistical analyses were carried out using Microsoft Excel (Microsoft Inc. 2013) and JMP 5.0.1 (SAS Institute Inc. 2001). Due to technical defaults 5 out of 240 total measurement scans were preliminarily excluded (all with kernel B80s, 4 with 0.6 slice thickness, 1 with 1.0 mm slice thickness) while 3  measurements with B70s kernel were partially incomplete, showing only L2. For further analysis 235 scans with 2882 out of 3024 data points were included (Table S1, Appendix). Precision errors were expressed by the calculation of the coefficients of variation. Coefficients of variation were calculated separately for each method and each body as well as a mean for vertebrae L1–L3 from the standard deviation of all BMD values during the measurement phase. We distinguished between midterm-precision (defined as the standard deviation calculated over one measurement period, 12 weeks) from long-term precision (which was defined as the standard deviation calculated over the total measurement period, 1.5 years), aiming for the lowest possible value. Accuracy is defined by the deviations of measured BMD from nominal values (mean of vertebrae L1–L3). For the asynchronous calibration methods deviations of mean BMD from standard ESP values were calculated for each ESP vertebral body and a mean over L1–L3 (Fig. 1a, b). To examine potential drifts in BMD a linear regression analysis was performed over time and annual rates of change (separately for the single vertebrae L1, L2, and L3 and averaged over three vertebrae L1–L3) were calculated for each method. 362 out of 382 data points were included for linear regression analysis with reconstruction kernels B70s, 0.6 mm and B40s, 1.0 mm slice thickness and monthly calibration with B40s, 1.0 mm slice thickness (Fig. 2a–h).

In the following section we present results of the BMD values averaged over all three vertebrae for the high-resolution protocol, kernel B70s, and the kernels B40s, B60s, B80s to investigate the impact of a sharp kernel with high resolution and a weak kernel with low radiation dose on the measurements.

## Results

### Accuracy and Precision of Phantom Data

Accuracy errors are depicted in Fig. 1a and b for trabecular and cortical bone compartments with asynchronous calibration (for HR-protocol and low dose protocols with B40s, B60s and B80s kernels) as well mid-term precision. Figures S3 and S4 (Appendix) depict deviations of measured BMD from nominal values for each ESP vertebra (L1, L2, and L3) individually. Accuracy is better for trabecular measurements than for cortical measurements for all calibration methods. BMD values in the trabecular compartments are mostly overestimated (+ 7.6 mg HA/cm^3^ with HR-protocol vs + 3.2 mg HA/cm^3^ with B40s-kernel, 1.0 mm slice-thickness), largest for kernels B60s, 0.6 mm slice thickness and B80s, 0.6 mm slice thickness (see Figs. S3 and S4, Appendix). In the cortical compartments, most BMD-values are consistently lower than expected, most for B40s with monthly calibration (− 355.8 mg HA/cm^3^) and least for B80s with 3.0 mm slice thickness (− 102.5 mg HA/cm^3^).

Mean coefficients of variation were lower with asynchronous (global) calibration for high resolution protocols (CV = 0.3% for trabecular bone, CV = 0.2% for cortical bone) as well as for low dose protocols (lowest for B80s, 0.6 mm slice thickness: CV = 1.4% for trabecular bone and B40s, 0.6 mm slice thickness: CV 0.4% for cortical bone). CV results for each ESP vertebra individually are depicted in Tables S2 a and b (Appendix).

Results for long-term precision were better for asynchronous than for simultaneous calibration both for trabecular and cortical bone compartments. Lowest long-term precision with asynchronous calibration was measured with B70s kernel for high resolution protocol (0.4 for trabecular bone vs. 1.1 for cortical bone) and B80s, 1 mm slice thickness (1.8 for trabecular bone) as well as B40s, 1 mm slice thickness (1.5 for cortical bone compartments) with low dose protocols.

### Linear Regression Analysis

Using a linear regression analysis relative annual change rates were calculated separately for the single vertebrae L1, L2, and L3 for each reconstruction kernel and slice thickness (Tables S3 a, b). Mean annual change rates are summarized in Tables 2 and 3, while drifts over time are depicted in Fig. 2a–h for simultaneous and global calibration for both bone compartment. Mean annual change rates for ESP vertebrae L1–L3 in trabecular bone were consistently < 2.0%, in cortical compartments mean annual change rates did not exceed 3.0%.

**Table 2:** Mean annual change rate (in %) (for ESP vertebrae L1–L3) for trabecular bone with simultaneous and asynchronous (global) calibration methods for reconstruction kernels B70s, 0.6 mm and B40s, 0.6 mm, 1.0 mm, 3.0 mm slice thickness) and monthly calibration for B40s, 1.0 mm slice thickness

	Trabecular bone			
	Sim. Cal		Asynchr. Cal	
	Annual change rate	*p*	Annual change rate	*p*
B70s, 0.6 mm	− 1.54	***	− 0.12	ns
B40s, 0.6 mm	0.20	ns	0.78	ns
B40s, 1.0 mm	0.68	ns	− 0.78	ns
B40s, 3.0 mm	− 0.81	ns	− 0.91	ns
B40s, 1.0 mm (monthly)	–		0.66	ns

*p* = *p*-values (ns: *p* ≥ 0.05, °: *p* ≤ 0.05, *p ≤ 0.01, **p ≤ 0.001, ***p ≤ 0.0001)

**Table 3:** Mean annual change rate (in %) (for ESP vertebrae L1–L3) for cortical bone with simultaneous and asynchronous (global) calibration methods for reconstruction kernels B70s, 0.6 mm and B40s, 0.6 mm, 1.0 mm, 3.0 mm slice thickness) and monthly calibration for B40s, 1.0 mm slice thickness

	Cortical bone			
	Sim. Cal		Asynchr. Cal	
	Annual change rate	*p*	Annual change rate	*p*
B70s, 0.6 mm	− 0.85	**	− 0.20	**
B40s, 0.6 mm	0.15	ns	− 0.50	***
B40s, 1.0 mm	− 0.28	ns	− 0.38	*
B40s, 3.0 mm	− 2.71	ns	− 1.53	ns
B40s, 1.0 mm (monthly)	–		0.63	ns

*p* = *p*-values (ns: *p* ≥ 0.05, °*p* ≤ 0.05, *p ≤ 0.01, **p ≤ 0.001, ***p ≤ 0.0001)

In trabecular compartments averaged HR-BMD remains stable for global (− 0.1%/year, ns) but not simultaneous calibration (− 1.5%/year, *p* < 0.001). No significant changes could be detected for averaged low dose BMD (− 0.9 to + 0.8%/year) for either calibration method. In cortical compartments, no or only unsystematic small negative drifts occur (HR: − 0.9 to − 0.2%/year, low dose: − 2.7 to 0.6%/year).

## Discussion

Our studies were intended to test the long-term reproducibility of vertebral BMD measurements with QCT using simultaneous and asynchronous calibration methods for a variety of different measurement and reconstruction protocols. These included a high-resolution protocol with enhanced spatial resolution but also larger radiation exposure and a low resolution and low dose protocol with reduced exposure, each compared to standard dose QCT. Our results document very good levels of precision and accuracy for both asynchronous and simultaneous calibrations with small but consistent advantages for asynchronous calibration: reproducibility (precision) was found to be better both for trabecular and cortical bone and for both the HR and low dose protocols and even for a single global calibration no systematic drift over time was observed, documenting that CT scanners are stable enough for longitudinal studies in clinical practice. While no drift problem was observed in our single scanner study, malfunctioning equipment can be encountered. For this reason, monthly calibration checks are recommended if simultaneous calibration is not performed.

To gain comparable, clinically adequate densitometric QCT measurements, a certain precision has to be achieved. Precision is usually quoted as a coefficient of variation and varies greatly between phantom (in vitro) studies and patient (in vivo) studies. In vivo studies with simultaneous calibration coefficients of variation range between 1 and 1.5% for trabecular and 2.5–3.0% for cortical bone [19], while in vitro studies have shown coefficients of variation between 1.0 and 4.0% regarding short term precision [20]. To our knowledge there are yet no studies investigating long-term precision with asynchronous calibration methods using a great number of different reconstruction protocols. For clinical practice in order to monitor annual BMD changes over time during follow-up examinations precision of DXA bone densitometry should be approximately 2.0% as stated by the International Society for Clinical Densitometry and for QCT [21], because of its greater responsiveness, somewhere higher precision errors can be tolerated. In our study, coefficients of variation were lower for asynchronous than for simultaneous calibration methods both for trabecular and cortical bone. The fact that asynchronous calibration yields a better precision indicates that a simultaneous calibration adds additional variability to the calculation of BMD. This indicates that a measurement without simultaneous calibration is not only feasible but might even be more precise. The higher precision error of simultaneous calibration might be due to the small size of the rods within the calibration phantom which limit averaging of voxels.

We also compared two different CT protocol: an HR protocol for optimized image quality as needed for the assessment of spinal trabecular bone structure and a low dose protocol targeting bone QCT densitometry that does not need the exposure levels of standard QCT protocols. We observed, not surprisingly, that precision errors were consistently lower for the high-resolution protocol (< 1.0%) than for low dose protocols both in trabecular and cortical bone. Best precision was found for the high-resolution protocol in asynchronous mode. For low resolution, protocols precision errors for asynchronous calibration were consistently approximately around 2%. While for L2 and L3 vertebrae precision errors were mostly below 3.0% a broader distribution could be found for L1 in soft kernel B40s, especially in trabecular bone compartments (CV_synchr_. = 7.8% and CV_asynchr._ = 7.4% both for B40s, 0.6 mm). Interestingly greater slice thicknesses (e.g., 3.0 vs. 1.0 mm) did not automatically correlate with the level of precision errors. However, smaller anatomic structures (e.g., endplates) were more easily detected using smaller slice thicknesses (e.g., 0.6, 1.0 mm) thus making imaging processing more practicable. Considering the criteria of low precision errors, sufficient image quality, and low radiation dosage, we suggest the use of B40s kernels with 0.6 or 1.0 mm slice thickness with asynchronous calibration to be the best choice for clinical practise.

Another aspect for fracture prediction is accuracy, which is defined as the degree to which the measured results deviate from the ‘true’ values [22]. To effectively diagnose osteoporosis and to assess individual fracture risks accuracy errors of 10–15 mg/HA cm^3^ (approximately ¼ to ½ standard deviations of the population variety) are tolerable [23]. Interestingly, earlier studies have additionally suggested that simultaneous calibration may cause additional phantom-induced bias due to a potential X-ray beam hardening. This bias could affect accuracy errors. However, since accuracy errors are substantially larger than precision error this additional accuracy error component might not be clinically relevant [11]. In our study, the BMD of cortical bone was systematically underestimated for ESP’s vertebral bodies L1 and L2. A difference in underestimation between different reconstruction protocols could not be identified. Reasons for underestimation could relate to partial volume effects and problems with threshold segmentation. The ESP’s cortical thickness differences from thin cortex L1, medium thickness L2 to thick cortex L3 combined with low spatial resolution and high slice thickness could have led to a massive underestimation of cortical bone BMD (Figs. S2 and S4, Appendix). Segmentation problems in the cortical region are widely cited in literature reviews. Possible solutions such as the measurement in vivo of bone mineral content for QCT and porosity for HRpQCT [7, 24] are being discussed. Regarding deviation of mean BMD from standard ESP values for trabecular bone for asynchronous calibration our results show low deviations and thus good accuracy for high resolution protocols and kernels B40s (slice thickness 0.6, 1.0, and 3.0 mm) and are therefore, recommended. Nevertheless, a small but systematic overestimation appears, stronger for the high-resolution protocol (dBMD_asynchr_. = 7.6 mg/HA cm^3^) than for the low dose protocol B40s (dBMD_asynchr._ = 2.9–3.9 mg/HA cm^3^). Since the ESP was placed in the middle of the CT-tube and the calibration phantom below the middle, mode dependent field inhomogeneities might play a role in the explanation of these deviations. Harder kernels 60 s and B80s (especially with slice thickness 0.6 mm) have significantly higher deviations (e.g., dBMD_asynchr_. = 29.3 mg/HA cm^3^, B80s, 0.6 mm) which we attribute to higher noise levels. Therefore, these kernels appear to be less suitable for long-term measurements with asynchronous calibration, at least in a low dose configuration.

As trabecular bone loss is known to show the earliest, this area is an important site to effectively diagnose and monitor fracture risk [25]. Mean annual change rates both for cortical and trabecular bone with both synchronous and asynchronous calibration were consistently low (mostly < 1.0% p.a.) showing good scanner constancy. In our studies, inconsistent drifts of low magnitude could be shown, indicating a very stable performance of the CT-scanner over one year. Based on these finding, it would be possible to use asynchronous BMD measurements for longitudinal assessment of change in BMD. No systematic advantage of simultaneous over global calibration could be found. The majority of significant annual relative change rates could be identified for ESP vertebra L3 for trabecular bone as well as for cortical bone (Tables S3 a and b in the Appendix). Reasons for this observation remain yet unclear but as annual change rates were max. 2.0%, we argue that those phenomena are not clinically relevant. Moreover, we identified significantly lower mean BMD-values over time for trabecular bone with B40s-kernel with 1 mm slice thickness with simultaneous calibration on measurement day No. 393 (see Fig. 2b) which lied outside of the 3 SD margin. A re-examination of these data points did not show a specific reason for such low BMD values as no segmentation or calibration errors could be found. As no systematic drifts could be identified over a period of 400 days, a good performance of our scanner can be reported. For a clinical setting, if global calibration is chosen, quality assurance measures to monitor drifts or instrument failures comprising quantitative phantom measurements should be applied at regular time intervals (e.g., monthly). Established methods (e.g., Shewhart charts [26]) to recognize relevant malfunction should be implemented.

Our study has several strengths. The comparison of synchronous versus asynchronous was performed at several kernel settings for both mid- and long-term precision. Also, the accuracy as well as long-term scanner stability were assessed in these settings. The inclusion of a high resolution and low dose protocols spans the range of reasonable radiation exposure levels for QCT. More detail can be found in a dissertation published on this topic [27]. This study also has some limitation. While a technical assessment of this breadth could not be performed in patients in vivo (due to ethical aspects related to radiation exposure) the study was limited to the use of one type of CT scanner and one type of calibration phantom at one single study-centre. Generalizability to other scanners would have to be shown. However, we maintain that the documentation that asynchronous calibration yields at least equivalent performance of precision compared to simultaneous calibration should not be dependent on scanner type—as long as stability is assured by monthly monitoring.

To transfer the findings of our study onto clinical settings, a number of external influences have yet to be investigated. Pompe et al. stress the fact that intravenous contrast agents do have a significant effect on BMD assessment using asynchronous calibration techniques [28]. The effect of contrast agents has not been investigated in this study. Moreover, Johannesdottir et al. outlines the dependency of CT scanner types on phantom calibration [6] while studies by Budoff et al. have shown that phantom-less BMD measurements acquired with a variety of 14 different CT scanners are equally accurate [29]. Budoff et al. indicate that thoracic BMD and lumbar BMD highly correlate in simultaneous coronary CT imaging while showing a gradual decrease of BMD from T1 to L3 [30]. These findings should be confirmed in further ongoing studies using asynchronous calibration methods in the thoracic region.

In conclusion, with state-of-the art CT technology data derived with synchronously and asynchronously calibrated methods can be equally used for vertebral BMD measurements. Provided that regular phantom calibration methods confirm stability for a given clinical scanner asynchronous calibration may even offer advantages in precision and clinical applicability.

## Supplementary Information

Below is the link to the electronic supplementary material.

Supplementary file1 (DOCX 243 KB)
