# Supplementary material for: Long-Term Reproducibility of BMD-Measurements with Clinical QCT Using Simultaneous and Asynchronous Calibration Methods and Different Measurement and Reconstruction Protocols
Source: Calcif Tissue Int. 2024 Oct 16;115(5):552–61. doi: 10.1007/s00223-024-01303-3 (PMC11531420; doi:10.1007/s00223-024-01303-3)
Supplement: Supplementary file 1 — Supplementary file1 (DOCX 243 KB) [file 223_2024_1303_MOESM1_ESM.docx]

**Appendix**

**Figures**


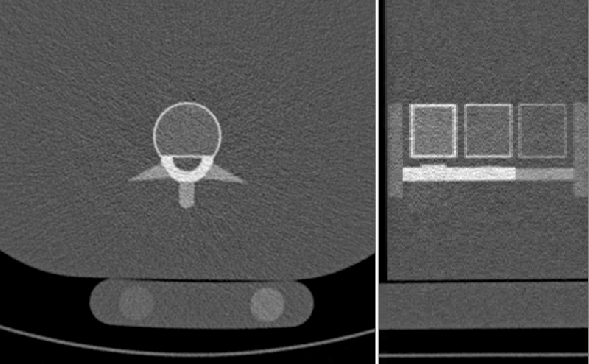


**Figure S1** Setup with ESP placed on the BDC phantom (left: axial view, right: sagittal view), low-dose scan with B40s-kernel with 1.0 mm slice thickness, date of measurement: 05.09.2017


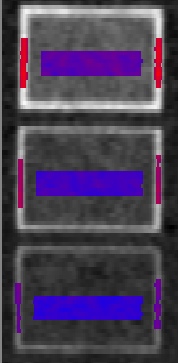


**Figure S2** QCT scans in sagittal axis of ESP after completed threshold segmentation using StructuralInsight (from left to right: L1, L2, L3). Cortical bone (only vertical part of the cortex, regions of analysis marked red and violet) and trabecular bone (regions of analysis marked blue) can now be analysed separately, low-dose scan with B40s-kernel with 1.0 mm slice thickness, date of measurement: 05.09.2017

**Figure S3** deviation of mean BMD from nominal BMD values (according to the QRM acceptance-test) for trabecular bone with asynchronous (global) calibration for all reconstruction kernels and monthly calibration for B40s, 1.0 mm slice thickness

**Figure S4** deviation of mean BMD from standard ESP values for cortical bone with asynchronous (global) calibration for all reconstruction kernels and monthly calibration for B40s, 1.0 mm slice thickness

**Tables**

**Table S1**

Summary of datasets scanned with synchronous calibration (N=number of complete measurements in total out of 240 , *=3 measurements are partially incomplete as only L2 was scanned by default, **= measurements are excluded due to technical faults)

| **Kernel** | **B70s** | **B40s** | | | **B60s** | | | **B80s** | | |
| --- | --- | --- | --- | --- | --- | --- | --- | --- | --- | --- |
| **Protocol type** | HRQCT | low dose | | | low dose | | | low dose | | |
| **Increment** | 0.3 | 0.3 | 1.0 | 3.0 | 0.3 | 1.0 | 3.0 | 0.3 | 1.0 | 3.0 |
| **Slice thickness** (in mm) | 0.6 | 0.6 | 1.0 | 3.0 | 0.6 | 1.0 | 3.0 | 0.6 | 1.0 | 3.0 |
| **N= 235** | 24^*^ | 24 | 24 | 24 | 24 | 24 | 24 | 20** | 23** | 24 |

**Table S2 a**

CV (in %) for trabecular bone with asynchronous and simultaneous calibration methods for all reconstruction and monthly calibration for B40s, 1.0 mm slice thickness

|  | **trabecular bone** | | | | | |
| --- | --- | --- | --- | --- | --- | --- |
|  | sim. cal. | | | asynchr. cal. | | |
|  | CV L1 | CV L2 | CV L3 | CV L1 | CV L2 | CV L3 |
| B70s, 0.6 mm | 1.69 | 0.71 | 0.69 | 0.66 | 0.47 | 0.58 |
| B40s, 0.6 mm | 7.82 | 3.31 | 2.49 | 7.39 | 2.93 | 1.64 |
| B40s, 1.0 mm | 4.48 | 3.56 | 2.78 | 4.06 | 2.67 | 1.53 |
| B40s, 3.0 mm | 3.16 | 3.28 | 2.60 | 3.83 | 2.34 | 1.86 |
| B40s, 1.0 mm (monthly) | - | - | - | 4.66 | 3.92 | 2.13 |
| B60s, 0.6 mm | 5.46 | 4.28 | 3.68 | 4.15 | 2.45 | 1.86 |
| B60s, 1.0 mm | 4.71 | 3.74 | 2.87 | 4.12 | 2.40 | 1.75 |
| B60s, 3.0 mm | 4.27 | 3.61 | 3.78 | 4.43 | 2.81 | 2.02 |
| B80s, 0.6 mm | 4.16 | 3.68 | 2.76 | 3.80 | 4.06 | 1.61 |
| B80s, 1.0 mm | 5.52 | 3.86 | 3.37 | 4.15 | 2.19 | 1.58 |
| B80s, 3.0 mm | 4.70 | 4.75 | 3.03 | 3.88 | 2.79 | 1.93 |

**Table S2 b**

CV (in %) for cortical bone with asynchronous and simultaneous calibration methods for all reconstruction and monthly calibration for B40s, 1.0 mm slice thickness

|  | **cortical bone** | | | | | |
| --- | --- | --- | --- | --- | --- | --- |
|  | sim. cal. | | | asynchr. cal. | | |
|  | CV L1 | CV L2 | CV L3 | CV L1 | CV L2 | CV L3 |
| B70s, 0.6 mm | 0.70 | 1.12 | 0.74 | 0.64 | 0.25 | 0.26 |
| B40s, 0.6 mm | 1.08 | 1.05 | 1.08 | 0.50 | 0.33 | 0.43 |
| B40s, 1.0 mm | 1.24 | 0.64 | 0.80 | 0.56 | 0.45 | 0.40 |
| B40s, 3.0 mm | 6.40 | 8.02 | 8.12 | 4.94 | 2.07 | 0.59 |
| B40s, 1.0 mm (monthly) | - | - | - | 1.34 | 1.42 | 1.38 |
| B60s, 0.6 mm | 1.87 | 1.97 | 2.01 | 0.50 | 0.42 | 0.44 |
| B60s, 1.0 mm | 1.81 | 1.85 | 2.14 | 0.44 | 0.51 | 0.40 |
| B60s, 3.0 mm | 4.21 | 2.67 | 2.25 | 5.30 | 2.62 | 0.86 |
| B80s, 0.6 mm | 3.50 | 5.72 | 2.83 | 0.79 | 0.35 | 0.41 |
| B80s, 1.0 mm | 2.58 | 2.19 | 4.36 | 0.87 | 0.38 | 0.43 |
| B80s, 3.0 mm | 3.17 | 2.88 | 3.28 | 4.08 | 1.51 | 2.18 |

**Table S3 a**

relative annual change rates of L1-L3 (in %) and p-values for all 10 reconstruction kernels and slice thicknesses for synchronous and asynchronous calibration methods for trabecular bone

|  | **trabecular bone** | | | | | | | | | | | |
| --- | --- | --- | --- | --- | --- | --- | --- | --- | --- | --- | --- | --- |
|  | sim. cal. | | | | | | asynchr. cal. | | | | | |
|  | L1 | p | L2 | p | L3 | p | L1 | p | L2 | p | L3 | p |
| B70s, 0.6 mm | -2.42 | * | -0.98 | * | -1.24 | *** | 0.18 | ns | -0.07 | ns | -0.31 | ° |
| B40s, 0.6 mm | -1.29 | ns | 2.59 | ns | -0.70 | ns | 2.68 | ns | 1.21 | ns | -1.55 | ° |
| B40s, 1.0 mm | 1.49 | ns | 1.72 | ns | -1.16 | ns | -2.48 | ns | 1.94 | ns | -1.79 | * |
| B40s, 3.0 mm | -1.52 | ns | 1.34 | ns | -2.25 | ns | -0.97 | ns | 0.01 | ns | -1.78 | ° |
| B40s, 1.0 mm (monthly) | - | - | - | - | - | - | -2.22 | ns | 4.13 | ° | 0.08 | ns |
| B60s, 0.6 mm | -2.77 | ns | -0.03 | ns | -1.55 | ns | -2.16 | ns | -0.37 | ns | -1.41 | ns |
| B60s, 1.0 mm | -2.13 | ns | 2.08 | ns | -0.18 | ns | -2.30 | ns | 1.40 | ns | -1.80 | ° |
| B60s, 3.0 mm | -2.69 | ns | -1.97 | ns | -2.76 | ns | 0.21 | ns | 0.83 | ns | -1.47 | ns |
| B80s, 0.6 mm | -3.22 | ns | -1.64 | ns | -3.58 | * | -0.79 | ns | 0.94 | ns | -1.00 | ns |
| B80s, 1.0 mm | -3.48 | ns | 0.99 | ns | -1.41 | ns | -1.99 | ns | 1.76 | ns | -1.43 | ° |
| B80s, 3.0 mm | -0.52 | ns | 2.79 | ns | -0.03 | ns | -2.45 | ns | -0.87 | ns | -2.04 | ° |

p=p-values (ns: p≥0.05, °: p≤0.05, *: p≤0.01, **: p≤0.001, ***: p≤0.0001)

**Table S3 b**

relative annual change rates of L1-L3 (in %) and p-values for all 10 reconstruction kernels and slice thicknesses for synchronous and asynchronous calibration methods for cortical bone

|  | **cortical bone** | | | | | | | | | | | |
| --- | --- | --- | --- | --- | --- | --- | --- | --- | --- | --- | --- | --- |
|  | sim. cal. | | | | | | asynchr. cal. | | | | | |
|  | L1 | p | L2 | p | L3 | p | L1 | p | L2 | p | L3 | p |
| B70s, 0.6 mm | -0.97 | * | -0.69 | * | -0.88 | ° | -0.01 | ns | -0.29 | * | -0.30 | ° |
| B40s, 0.6 mm | -0.45 | ns | 0.11 | ns | -0.12 | ns | -0.42 | ns | -0.46 | ** | -0.61 | ** |
| B40s, 1.0 mm | -0.92 | ns | 0.21 | ns | -0.15 | ns | -0.20 | ns | -0.38 | ns | -0.57 | ** |
| B40s, 3.0 mm | -0.32 | ns | -3.98 | ns | -3.82 | ns | -2.16 | ns | -1.63 | ns | -0.79 | ° |
| B40s, 1.0 mm  (monthly) | - | - | - | - | - | - | 0.60 | ns | 0.10 | ns | 1.18 | ns |
| B60s, 0.6 mm | -1.04 | ns | -0.83 | ns | -0.80 | ns | -0.54 | ° | -0.47 | * | -0.62 | ** |
| B60s, 1.0 mm | -1.12 | ns | -0.28 | ns | -0.59 | ns | -0.72 | ** | -0.40 | ° | -0.71 | ** |
| B60s, 3.0 mm | -1.93 | ns | -1.29 | ns | -0.77 | ns | 2.34 | ns | 1.16 | ns | -0.96 | ° |
| B80s, 0.6 mm | -3.10 | ns | -4.40 | ns | -3.08 | ° | -0.34 | ns | -0.27 | ns | -0.52 | * |
| B80s, 1.0 mm | 0.30 | ns | 1.15 | ns | 2.92 | ns | -0.80 | ° | -0.39 | ° | -0.57 | * |
| B80s, 3.0 mm | 2.80 | ° | 2.01 | ns | 2.54 | ns | 1.21 | ns | 0.10 | ns | -1.08 | ns |

p=p-values (ns: p≥0.05, °: p≤0.05, *: p≤0.01, **: p≤0.001, ***: p≤0.0001)
